# Cardiovascular magnetic resonance predictors of heart failure in hypertrophic cardiomyopathy: the role of myocardial replacement fibrosis and the microcirculation

**DOI:** 10.1186/s12968-021-00720-9

**Published:** 2021-03-08

**Authors:** Claire E. Raphael, Frances Mitchell, Gajen Sunthar Kanaganayagam, Alphonsus C. Liew, Elisa Di Pietro, Miguel Silva Vieira, Lina Kanapeckaite, Simon Newsome, John Gregson, Ruth Owen, Li-Yueh Hsu, Vassilis Vassiliou, Robert Cooper, Aamir Ali MRCP, Tevfik F. Ismail, Brandon Wong, Kristi Sun, Peter Gatehouse, David Firmin, Stuart Cook, Michael Frenneaux, Andrew Arai, Rory O’Hanlon, Dudley J. Pennell, Sanjay K. Prasad

**Affiliations:** 1 NIHR Cardiovascular Biomedical Research Unit, Royal Brompton Hospital, London, UK; 2 Department of Advanced Biomedical Sciences, University of Naples, Naples, Italy; 3 London School of Hygiene & Tropical Medicine, London, UK; 4 Advanced Cardiovascular Imaging Laboratory, National Heart, Lung, and Blood Institute, National Institutes of Health, Bethesda, Maryland USA; 5 Norwich Medical School, University of East Anglia, Norwich, UK; 6 King’s College London & Guy’s and St Thomas’ NHS Foundation Trust, London, UK; 7 National Heart Center, Singapore, Singapore; 8 Blackrock Clinic, Co. Dublin, Ireland; 9 Department of CMR, Royal Brompton Hospital, Sydney Street, Sydney, SW3 6NP UK

**Keywords:** Hypertrophic cardiomyopathy, Heart failure, Prognosis, Cardiovascular magnetic resonance, Fibrosis, Microvascular ischemia, Myocardial perfusion

## Abstract

**Introduction:**

Heart failure (HF) in hypertrophic cardiomyopathy (HCM) is associated with high morbidity and mortality. Predictors of HF, in particular the role of myocardial fibrosis and microvascular ischemia remain unclear. We assessed the predictive value of cardiovascular magnetic resonance (CMR) for development of HF in HCM in an observational cohort study.

**Methods:**

Serial patients with HCM underwent CMR, including adenosine first-pass perfusion, left atrial (LA) and left ventricular (LV) volumes indexed to body surface area (*i*) and late gadolinium enhancement (%LGE- as a % of total myocardial mass). We used a composite endpoint of HF death, cardiac transplantation, and progression to NYHA class III/IV.

**Results:**

A total of 543 patients with HCM underwent CMR, of whom 94 met the composite endpoint at baseline. The remaining 449 patients were followed for a median of 5.6 years. Thirty nine patients (8.7%) reached the composite endpoint of HF death (n = 7), cardiac transplantation (n = 2) and progression to NYHA class III/IV (n = 20). The annual incidence of HF was 2.0 per 100 person-years, 95% CI (1.6–2.6). Age, previous non-sustained ventricular tachycardia, LV end-systolic volume indexed to body surface area (LVESVI), LA volume index ; LV ejection fraction, %LGE and presence of mitral regurgitation were significant univariable predictors of HF, with LVESVI (Hazard ratio (HR) 1.44, 95% confidence interval (95% CI) 1.16–1.78, p = 0.001), %LGE per 10% (HR 1.44, 95%CI 1.14–1.82, p = 0.002) age (HR 1.37, 95% CI 1.06–1.77, p = 0.02) and mitral regurgitation (HR 2.6, p = 0.02) remaining independently predictive on multivariable analysis. The presence or extent of inducible perfusion defect assessed using a visual score did not predict outcome (p = 0.16, p = 0.27 respectively).

**Discussion:**

The annual incidence of HF in a contemporary ambulatory HCM population undergoing CMR is low. Myocardial fibrosis and LVESVI are strongly predictive of future HF, however CMR visual assessment of myocardial perfusion was not.

## Introduction

Patients with hypertrophic cardiomyopathy (HCM) are at risk of heart failure (HF) [1–3] and the annual mortality in these patients is ten-fold higher than the general HCM population. Patients with HCM and HF have a high risk of death from both progressive pump failure and sudden cardiac death (SCD) [4, 5].

There is limited understanding of the mechanisms underlying development of HF in HCM. Two areas of active interest are the presence of myocardial replacement fibrosis and abnormalities in the microcirculation. Patients with HCM often have abnormal myocardial perfusion [6] and recurrent bouts of ischaemia are hypothesised to lead to myocardial fibrosis and development of systolic dysfunction [7, 8]. Replacement myocardial fibrosis has been shown to predict SCD in HCM [9, 10] but its relationship to HF is not clear.

Cardiovascular magnetic resonance (CMR) allows accurate assessment of left ventricular (LV) volumes and function, identification and quantification of myocardial fibrosis using late gadolinium imaging (LGE), and assessment of myocardial perfusion [11]. We used CMR to assess potential mechanistic drivers of HF, in particular, the role of myocardial replacement fibrosis and microvascular ischemia. We hypothesised that the degree of myocardial ischemia and replacement fibrosis would predict future HF and aimed to assess whether there was added value in routine perfusion imaging for the identification of HCM patients at high risk of HF.

## Methods

### Patient recruitment

Consecutive patients with a diagnosis of HCM seen in the inherited cardiomyopathy service or referred to the Royal Brompton Hospital for CMR between December 2003 and April 2013 were prospectively recruited into a registry. CMR analysis of perfusion using a visual score was performed retrospectively. All patients provided written informed consent for inclusion in the study. The study was approved by the local institutional ethics committee.

All patients met the American Heart Association criteria for diagnosis of HCM, defined as a wall thickness of 15mm or greater, or 13–14mm if there was a first degree relative with HCM, not explained by another cardiac or systemic disease causing abnormal loading conditions[12].

CMR first pass perfusion was initially performed in a pilot HCM cohort and after an initial safety phase, recruitment was ramped. Based on data from nuclear imaging [13] and this safety data, dynamic LV outflow tract (LVOT) obstruction was not a contraindication for intravenous adenosine infusion.

We excluded patients who met our HF definition at baseline, known metabolic diseases causing a HCM phenocopy, e.g., Anderson-Fabry and Noonan’s syndrome, prior surgical myectomy or alcohol septal ablation, and patients with contra-indications to CMR, including presence of an implantable cardioverter defibrillator (ICD) or pacemaker. Patients with an estimated glomerular filtration rate less than 30 ml/min/1.73 m^2^ were not given gadolinium contrast. Patients with known significant coronary artery disease, defined as > 70 % stenosis in an epicardial artery of 2mm or greater were excluded from analysis.

The predefined primary endpoint was a new major HF event defined as a composite of HF death, cardiac transplantation for HF and progression to New York Heart Association class III/IV. HF death was defined as death associated with unstable, progressive deterioration of pump function or symptoms associated with HF. We additionally collected episodes of HF hospitalization, defined as an unplanned admission of greater than 24 hours with new or worsening signs of HF, including radiographic evidence of pulmonary edema and/or need for intravenous diuretics [14].

The CMR-LGE component of this study was part of a previous LGE outcomes study: 185 patients were included in a previous outcomes analysis using a composite endpoint looking at major adverse cardiovascular events including HF [10]. In the present study, we additionally evaluated myocardial perfusion and present extended follow up.

### CMR protocol

CMR scans were performed on a 1.5 T CMR scanner (Sonata/Avanto, Siemens Healthineers, Erlangen, Germany) using a standardized protocol as previously described [10]. Patients were asked to abstain from dipyridamole, aminophylline, beta blockers or rate-limiting calcium channel antagonists for 48 hours and caffeine-containing substances for 24 hours prior to imaging.

Myocardial first-pass perfusion imaging was performed using a saturation-recovery prepared dual-sequence approach with center-out hybrid echoplanar imaging and the following typical parameters: fat saturation pulse, composite 90° saturation preparation pulse for each slice, 28° readout pulse, repetition time 5.1 ms, echo time 1.1ms, echo train length 4, field of view 360 × 288 mm, base resolution 160 × 160, slice thickness 8 mm. Shimming was performed to ensure maximum magnetic field homogeneity and minimise off-resonance effects. Test images were taken to identify any artefacts. Adenosine was infused at 140 mcg/kg/min for 4 minutes and symptoms, heart rate and blood pressure were monitored. At peak hyperaemia, a 0.1 mmol/kg bolus of gadolinium contrast (Magnevist or Gadovist, Bayer-Schering, Berlin, Germany) was rapidly injected, followed by a saline bolus. Three short axis images were acquired every cardiac cycle for a total of 30 cycles at peak hyperaemia.

LGE imaging was performed using a spoiled gradient-echo segmented k space breath hold sequence in long and short axis planes, 10 min after injection of gadolinium contrast. Inversion times were optimised to null normal myocardium and images were repeated in 2 separate phase-encoding directions to allow exclusion of artifact. Typical sequence parameters were TE 3.1ms, TR 7ms, 8mm slice thickness, 25 degrees flip angle, field of view 380 × 310m m, 25 phase encodes per cardiac cycle. After 20 minutes, rest perfusion imaging was carried out using the same slice positions and gadolinium bolus preparation.

### CMR image analysis

Image analysis was performed by experienced operators blinded to clinical outcome. Biventricular volumes and mass were measured using dedicated semi-automated software (CMRtools, Cardiovascular Imaging Solutions, London, UK) and indexed to body surface area (BSA). Mitral regurgitation was characterized by visual assessment and calculation of the regurgitant fraction using stroke volume difference between the LV and right ventricles. If LGE was present, the extent was quantified from the short axis stack, using commercially available software (cvi42, Circle Cardiovascular Imaging, Calgary, Alberta, Canada). The endocardial and epicardial borders were manually contoured and an area of remote myocardium free of replacement fibrosis and artifact was defined. Fibrosis was quantified using the “full width half maximum” (FWHM) technique and expressed as a percentage of total left ventricular mass, %LGE [15].

Left atrial (LA) area and length were recorded from the 2- and 4- chamber long axis images at end-ventricular systole, just prior to the opening of the mitral valve. The LA length was measured from the midpoint of the mitral valve annulus plane to the top of the LA in both planes. LA volume was calculated as follows [16]:

$$LA\;volume\;\left(ml\right)\;=\;\frac{8\left(A_{2CH}\right)\left(A_{4CH}\right)}{3\pi L}$$

where A_2CH_ is the area in the 2-chamber view, A_4CH_ is the area in the 4-chamber view and L is the shorter of the two LA length measurements.

### CMR visual perfusion scoring

An inducible perfusion defect was considered present if a subendocardial or transmural area of signal hypointensity was visualised and persisted for 3 frames or more after the first arrival of LV myocardial contrast on stress images but not in corresponding rest images. These were distinguished from dark rim artefact due to extent and persistence of the defect. Papillary muscles were excluded from perfusion assessment. In any patient where there was disagreement regarding presence of a perfusion defect, final decision was made by consensus. Two experienced operators blinded to clinical outcome (CER, MSV) assessed perfusion used a summed difference score (SDS) [17, 18] using the American College of Cardiology (ACC)/American Heart Association (AHA) 17 segment model [19], excluding the cardiac apex (segment 17). Segments with LGE enhancement were not excluded from analysis. Each segment was scored at stress and rest as follows: 0- no defect, 1-inducible perfusion defect < 50 % of wall thickness, 2- inducible perfusion defect > 50 % of wall thickness [17, 18]. The rest score was subtracted from the stress score to give the SDS.

### Validation of visual perfusion score

In a subset of patients, myocardial blood flow (MBF) was quantitated at rest and at peak stress, allowing calculation of the myocardial perfusion reserve index (MPRI) as stress MBF/rest MBF according to previously described methods [20, 21].

### Definition of end points

Events were adjudicated by an independent committee blinded to CMR results. Mortality status was checked at 6 monthly intervals via the UK National Strategic Tracing Service. Cause of death was defined following detailed review of medical records, death certification, postmortem data and communication with the patients’ primary care physicians and cardiologists. Patients were followed up by telephone and/or postal questionnaire at 6-month intervals and medical records from primary and secondary care were obtained every 6 months.

### Statistical analysis

Baseline characteristics were presented as frequency (percentage) for categorical data and mean (standard deviation, SD) for continuous data unless otherwise stated. The correlation between the SDS score and quantitative MPRI was assessed using Pearson correlation coefficient. Intra- and inter- operator agreement were assessed using Pearson correlation coefficient and Bland Altman analysis, reported as mean difference ± SD of the differences. Continuous variables were dichotomised into groups for generation of Kaplan-Meier survival curves. Kaplan-Meier survival curves were compared using the log-rank test. Univariable Cox proportional hazards models were used to test the association between baseline covariates and the endpoint. Variables which were significant in the univariable analysis were included in multivariable analyses. Multivariable Cox proportional hazards models were used to test the independence of the identified predictors of interest. For the combined HF endpoint, data were censored after the first component of the composite endpoint. Results are presented as hazard ratios (95 % confidence intervals). A two-tailed p-value < 0.05 was considered significant. Incidence of new HF was defined as the number of new cases meeting the HF definition over the follow up period, divided by the total number of person-years of follow-up. Incidence was presented as an annualized rate per 100 person-years. Analyses were performed using Stata 14 (StatCorp, College Station, Texas, USA).

## Results

**Fig. 1:**
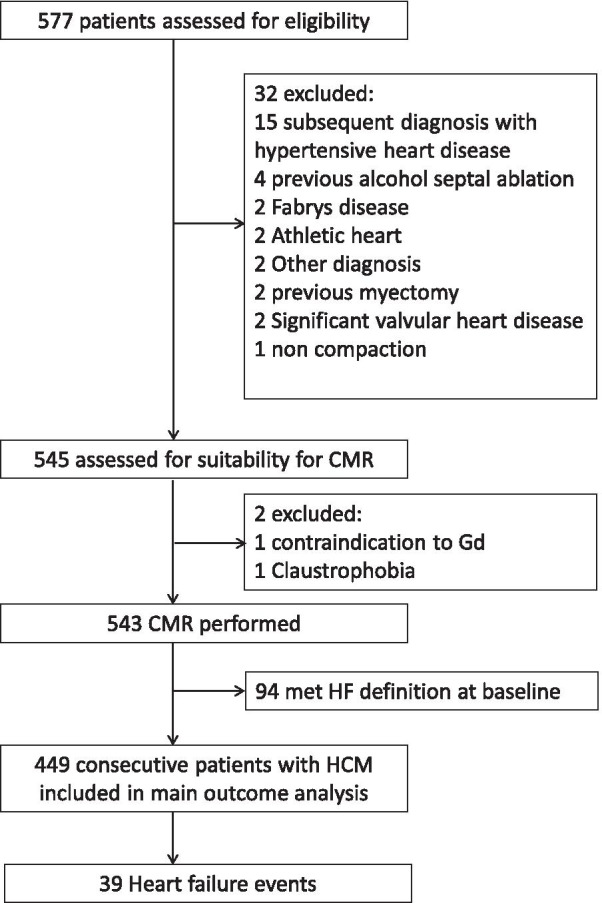
Identification of the study population

A total of 577 patients were assessed for eligibility, of which 34 were excluded (Fig. 1). Of the remaining 543, 94 patients met the HF endpoint at baseline (17%) and were excluded from further analysis, leaving a final study cohort of 449 patients with HCM. Patient characteristics are described in Table 1. Twenty four percent of patients were referred via the inherited cardiomyopathy clinic. A third of patients had resting LVOT obstruction.

**Table 1:** Baseline clinical demographics

	**New heart failure** **(n = 39)**	**No heart failure** **(n = 410)**
Age at baseline CMR, years	63 ± 9	59 ± 14
Age at diagnosis, years	57 ± 11	53 ± 15
Sex (% Male)	26 (67%)	308 (75%)
Apical variant	4 (10%)	66 (16%)
Atrial fibrillation	3 (8%)	8 (2%)
New York Heart Association Class		
I	20 (51%)	258 (63%)
II	19 (49%)	152 (37%)
III	0 (0%)	0 (0%)
LVOT obstruction > 30 mmHg	15 (41%)	148 (36%)
Family history of sudden death	7 (18%)	57 (14%)
Non sustained ventricular tachycardia	7 (18%)	45 (11%)
Syncope	9 (23%)	66 (16%)
Wall thickness ≥ 30 mm	4 (10%)	16 (4%)
CMR parameters (median, IQR)		
Wall Thickness, mm	20 (6)	19 (6)
LVEDVI, ml/BSA	66 (22)	67 (19)
LVESVI, ml/BSA	18 (10)	16 (9)
LVEF, %	73 (16)	76 (10)
LGE (% of total myocardial mass)	23 (20)	10 (20)
LGE (≥ 5%)	34 (87%)	295 (67%)
LAVI ml/BSA	65 (38)	53 (25)
Inducible perfusion defect	32 (82%)	344 (84%)
Perfusion summed difference score	14.4 (6.9)	12.8 (8.2)
Mitral regurgitation		
None	18 (46%)	254 (62%)
Mild	17 (44%)	127 (31%)
> Mild	4 (10%)	29 (7%)
Medications		
Beta blocker	25(64%)	250 (61%)
Calcium channel blocker	5 (13%)	70 (17%)
ACEi/ARB	3 (8%)	70 (17%)
Aspirin	11 (28%)	82 (20%)
Warfarin	7 (18%)	12 (3%)
Amiodarone	3 (8%)	4 (1%)
Co-morbidities		
Coronary artery disease	3 (8%)	41 (10%)
Chronic obstructive pulmonary disease	0 (0%)	7 (2%)
Hypercholesterolemia	11 (28%)	61 (15%)
Diabetes	7 (18%)	32 (8%)
Hypertension	10 (26%)	97 (24%)
Stroke	1 (3%)	3 (1%)

Data are presented as mean and standard deviation or number (% of total population) as appropriate

*LV* left ventricle, *EDVI* end diastolic volume index, *ESVI* end systolic volume index, *LGE* late gadolinium enhancement, *EF* ejection fraction, *ACEi* angiotensin converting enzyme inhibitor, *ARB* angiotensin II receptor blocker, *CMR* cardiovascular magnetic resonance, *LVOT* left ventricular outflow tract, *IQR* interquartile range, *LAVI* left atrial volume index

The majority of patients (n = 376; 84%) had a perfusion defect at peak adenosine stress. The majority of defects were subendocardial and in all coronary territories, suggestive of diffuse microvascular disease. Three patients had a perfusion defect corresponding to a coronary artery territory. Thirty six percent of patients had had recent coronary imaging (30% normal coronary angiogram, 5% coronary artery disease with prior revascularization and no significant stenoses on most recent imaging, 1% normal coronary computed tomography angiogram). There was no significant correlation between the severity of perfusion defect and the %LGE (r = 0.05, p = 0.34), however patients without a perfusion defect had a lower %LGE compared to those with a perfusion defect (median LGE 7.9% (IQR 1.1–16.3) vs 13.1% (3.8–24.7, p = 0.04).

The datasets generated and/or analysed during the current study are not publicly available due to ongoing research but are available from the corresponding author on reasonable request.

### Validation of visual perfusion score

**Fig. 2:**
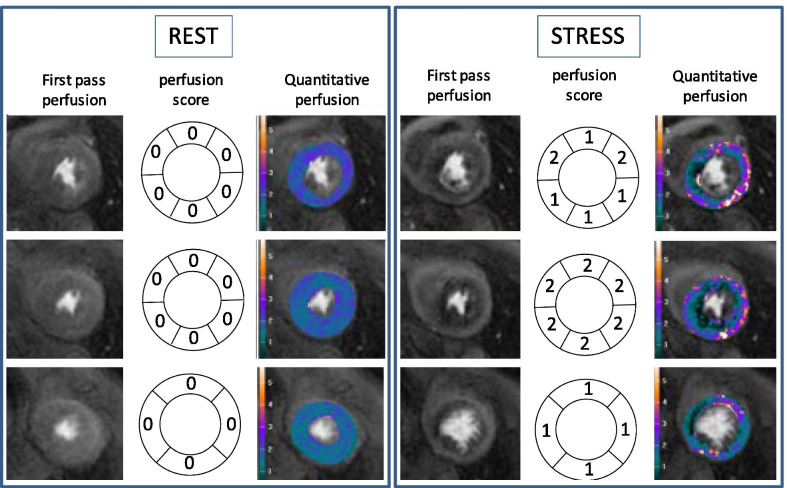
Assessment of perfusion defects in hypertrophic cardiomyopathy (HCM). Perfusion defects were assessed at rest (left panel) and adenosine stress (right panel). Stress perfusion defects were typically widespread throughout the 3 coronary territories. The endocardium was affected more than the epicardium. For visual assessment, perfusion was assessed using the American Heart Association (AHA) 17 segment model (excluding the apex) and scored as 0—no defect, 1—inducible perfusion defect < 50% of wall thickness, 2- inducible perfusion defect > 50% of wall thickness. The sum difference score (SDS) was calculated as the sum of the stress perfusion score minus the sum of the rest perfusion score. In this example, the SDS was (8 + 12 + 4)-(0 + 0 + 0) = 24. Quantitative perfusion was performed in a subgroup of patients and the myocardial perfusion reserve index (MPRI) compared to the SDS for validation

**Fig. 3:**
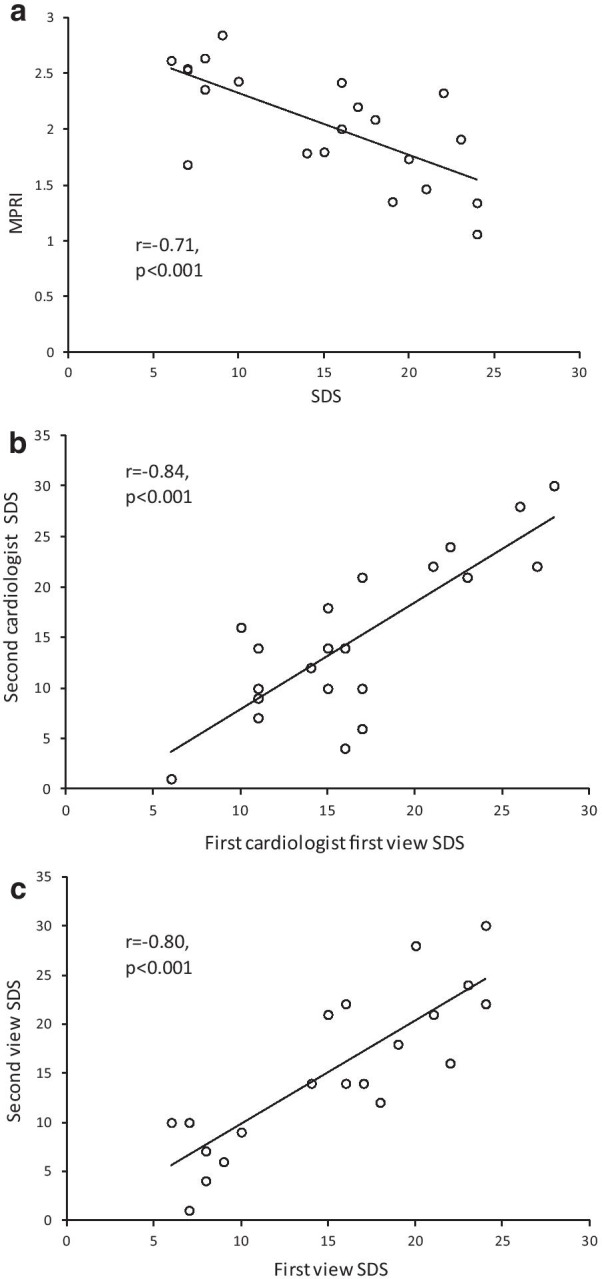
**a** Agreement between visual perfusion score (SDS) and quantitative MPRI in a subset of 21 HCM patients. **b** Intra-operator agreement for SDS score. **c** Inter-operator agreement for SDS score

The SDS and quantitative MPRI were assessed in a subset of 21 patients (Fig. 2). Intra-operator agreement for SDS was good (p = 0.84, p < 0.001) with a mean difference (+ SD) of 0.1 ± 4.2. Inter-operator agreement for SDS was also good (p = 0.80, p < 0.001) with a mean difference (+ SD) of 1.7 ± 4.7 (Fig. 3). The correlation between SDS and MPRI was reasonable (r = -0.71, p < 0.001, Fig. 3a).

### Heart failure events during follow up

The median follow-up time was 5.6 years (IQR 3.6–8.0 years). Events were censored at 8 years. Thirty-nine (8.7%) patients met the primary end point: 7 HF deaths (1.6%), 2 heart transplants (0.4%) and 30 with symptoms of NYHA class III/IV. This gave a calculated annual incidence of HF of 2.0 per 100 person-years, (95% confidence intervals 1.6–2.6 person-years). Of the patients who progressed to NYHA class III/IV, 14 had admission with decompensated HF requiring intravenous diuresis. Further details of the patients, divided by etiology of HF event (HF with reduced EF, HF with preserved EF, LVOT obstruction) are described in Table 2.

**Table 2:** Subclassification of heart failure events by etiology of heart failure

Heart failure event	HFpEF n = 13	HFrEF n = 20	LVOT obstruction n = 6
Heart failure death	2	4	1
Referral for cardiac transplantation	0	2	0
Progression to NYHA class III/IV	11	14	3
Decompensated HF requiring admission with diuresis	5	8	1

*HFpEF* heart failure with preserved ejection fraction, *HFrEF* heart failure with reduced ejection fraction, *NYHA* New York Heart Association

During follow up, 72 patients (16%) died, including 7 (1.6%) HF deaths and 3 (0.7%) sudden cardiac deaths. A total of 10 (2.2%) patients had myectomy and 58 (12.9%) had implantation of a cardiodefibrillator (ICD).

## Predictors of heart failure endpoint

**Fig. 4:**
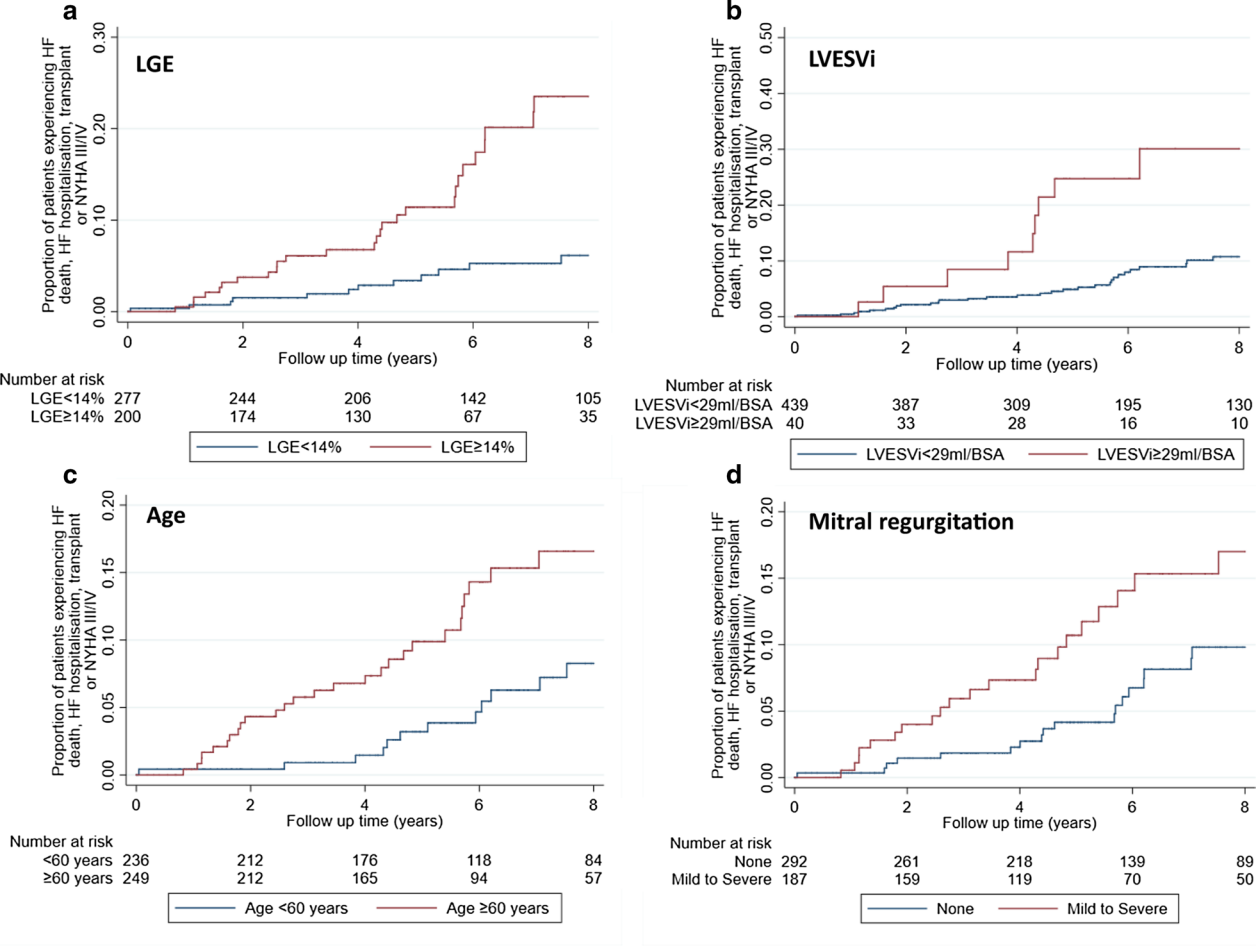
Kaplan–Meier curves for predictors of heart failure (HF) composite endpoint. Dichotomous cut offs for percent late gadolinium enhancement (%LGE), and left ventricular end-systolic volume index (LVESVI) and age are presented for clarity

There were eight predictors of the HF end point on univariable analysis (Table 3). These were age at baseline, previous non sustained ventricular tachycardia, LV end-systolic volume index (LVESVI), LA volume index (LAVI*)*, LV ejection fraction (LVEF), presence of LGE > 5% of total myocardial mass, %LGE and presence of mitral regurgitation. Presence or extent of inducible perfusion defect did not predict outcome (p = 0.16, p = 0.27 respectively). On multivariable analysis, four variables remained independently predictive (Table 4, Fig. 4).

**Table 3:** Univariable predictors of a heart failure event

	**HR (95% CI)**	**P**
Age (per 10 years)	1.32 (1.04, 1.67)	0.02
Age at diagnosis (per 10 years)	1.16 (0.93, 1.45)	0.18
Body surface area (kg/m^2^)	1.79 (0.41, 7.87)	0.44
Female	1.52 (0.78, 2.95)	0.22
Apical	0.47 (0.17, 1.32)	0.15
Atrial fibrillation	2.86 (0.88, 9.28)	0.08
LVOT gradient (≥ 30 mmHg at rest)	1.40 (0.74, 2.66)	0.3
Family history of sudden cardiac death	1.31 (0.60, 2.85)	0.5
Non sustained ventricular tachycardia	2.23 (1.02, 4.86)	0.04
Unexplained syncope	1.45 (0.69, 3.06)	0.33
Max wall thickness ≥ 30 mm	2.34 (0.83, 6.59)	0.11
CMR parameters		
Max wall thickness (mm)	1.03 (0.97, 1.09)	0.32
LVEDVI (per 10 ml/BSA)	1.19 (0.99, 1.45)	0.07
LVESVI (per 10 ml/BSA)	1.51 (1.23, 1.85)	<0.001
LAVI (per 10 ml/BSA)	1.11 (1.00, 1.22)	< 0.001
LVEF	0.95 (0.92, 0.98)	0.04
LGE (per 10%)	1.57 (1.27, 1.93)	0.001
Presence of LGE (≥ 5% of myocardial mass)^a^	3.99 (1.56, 10.22)	< 0.001
Perfusion defect	2.09 (0.74, 5.88)	0.004
Perfusion summed difference score	1.02 (0.98, 1.06)	0.16
Mitral regurgitation		
None	1	0.27
Mild	2.13 (1.10, 4.14)	0.03
Moderate/Severe	2.13 (0.72, 6.31)	0.27

Abbreviations as per Tables 1 and 2

^a^Patients with no LGE enhancement did not have any HF events, therefore < 5% LGE was used as the reference population to enable calculation of a HR

**Table 4:** Multivariable predictors of a new heart failure event

	**HR (95% CI)**	**P-value**
LVESVI (per 10 ml/BSA)	1.44 (1.16, 1.78)	0.001
Mitral regurgitation		
None	Reference group	0.02
Mild	1.94 (0.99, 3.81)	
Moderate/Severe	2.55 (0.84, 7.70)	
LGE (per 10%)	1.44 (1.14, 1.82)	0.002
Age (per 10 years)	1.37 (1.06, 1.77)	0.02

Abbreviations as per Tables 1 and 2

When divided by etiology of HF event, there were no significant predictors of a HF endpoint, however sensitivity was limited by low event numbers in the subgroups (Table 2). Predictors of all-cause death are listed in Table 5. Predictors of ICD implantation are described in Table 6.

**Table 5:** Univariable predictors of all-cause mortality

	HR (95% CI)	P
Age (per 10 years)	2.45 (1.85, 3.26)	< 0.001
LAVI (per 10)	1.10 (1.01, 1.21)	0.03
Unexplained syncope	2.06 (1.06, 4.01)	0.03
Hypertension	0.37 (0.14, 0.94)	0.04
Apical variant	0.12 (0.02, 0.91)	0.04

**Table 6:** Univariable predictors of implanted cardiodefibrillator (ICD) implantation

	OR (95% CI)	P
Non-sustained ventricular tachycardia	9.43 (4.84, 18.37)	< 0.001
Unexplained syncope	3.36 (1.74, 6.50)	< 0.001
Family history of sudden cardiac death	2.60 (1.31, 5.15)	0.006
Age (per 10 years)	0.80 (0.64, 0.99)	0.04

## Discussion

While management of sudden cardiac death has improved in HCM, HF remains an important cause of both morbidity and mortality. To date, there have been no prospective focused CMR studies that address predictors of HF in HCM. We report the contemporary incidence of HF in a large cohort of patients with HCM. The strongest predictors of future HF events were %LGE and LVESVI. The presence and severity of a visual perfusion defect as assessed by CMR did not predict HF.

The incidence of HF in our study was similar to earlier studies, which recruited patients in 1980s-2000s [5, 22, 23]. There has been no reduction in HF incidence despite contemporary management. Development and testing of novel therapies to reduce progression of HF therefore remains a key unmet need in HCM. Our data may guide patient selection and trial design for future trials of pharmacotherapy to prevent or delay development of HF in HCM.

### Predictors of heart failure in HCM

In our present study, we focused on potential mechanisms underlying the development of HF. As expected, co-morbidities that predispose to HF, including age, diabetes, hypercholesterolemia and mitral regurgitation, were predictive of HF outcomes on univariable analysis.

Replacement fibrosis increases ventricular stiffness and diastolic dysfunction. Progressive myocardial fibrosis may also directly result in reduced LVEF, as areas of extensive replacement fibrosis will no longer be able to contract. Over time, these changes may lead to LV failure and HF symptoms. LGE been shown to predict development of SCD in HCM and may allow better risk stratification than conventional scoring systems [9]. We demonstrated that %LGE is also a predictor of HF. HF in HCM is a multifactorial process with distinct phenotypes –hypokinetic evolution, restrictive physiology (preserved ejection fraction) and severe LVOT obstruction. It may be that %LGE has greater predictive value in the first two phenotypes compared to the latter. Our data was not powered to detect such differences but future multicenter studies such as HCM Registry may yield answers.

HCM is associated with structural abnormalities of the mitral valve including pathological elongation and leaflet thickening [24]. Mitral regurgitation was predictive of HF, independent of LVESVI. Whether this is causative, resulting from the inability of the small volume HCM heart to deal with the increased regurgitant volume, or due to association with LVOT obstruction, remains a target for future research. Of note, resting LVOT obstruction was not predictive of HF in our cohort, although we did not have complete data on latent LVOT obstruction which is an important limitation.

#### Perfusion imaging in HCM

Prior work in HCM has demonstrated that inducible perfusion defects are present in over half of patients and that these are typically global and subendocardial, representing widespread abnormalities in the microcirculation [25, 26]. This is in keeping with pathology studies demonstrating arteriolar dysplasia and hypertrophy [27]. Interestingly, impaired myocardial oxygenation was seen in carriers of HCM mutations prior to development of LV hypertrophy, suggesting the microcirculation may be affected early in the disease process [28]. Similar to previous work [26], we demonstrated that the presence, but not the extent of abnormal myocardial perfusion, was associated with higher %LGE. We have previously demonstrated that a subgroup of patients with HCM have regions of myocardium where the stress myocardial blood flow is lower than that at rest, which is likely to result in myocardial ischemia [21]. This is likely to be one of the drivers of myocardial fibrosis.

Perfusion imaging is currently not recommended for risk stratification in HCM [29]. While positron emission tomography (PET) studies in a small cohort of patients (n = 51) demonstrated that myocardial blood flow was a powerful independent predictor of death [7] and HF [8], larger studies using thallium single photon emission tomotraphy (SPECT) imaging showed no relationship between perfusion abnormalities and outcome [30]. It is possible that the strong predictive value of PET may reflect a composite measure of myocardial perfusion and fibrosis, since perfusion will be reduced in areas of replacement fibrosis [8]. It may also suggest that the development of HF in HCM is due to the primary disease process rather than propensity to recurrent ischemia. Unlike PET, visual CMR perfusion and SPECT rely on a relative, rather than an absolute assessment of perfusion. Since perfusion abnormalities in HCM are typically diffuse [31], absolute measures of perfusion may be required for accurate assessment. There was moderate agreement between the visual SDS score and quantitative CMR perfusion and the predictive value of myocardial perfusion may have been different had we used a fully quantitative perfusion method. Quantitative perfusion CMR requires specialised sequences and lengthy analysis time which would be unfeasible for use in routine clinical practice.

#### Incidence of heart failure compared to earlier studies

Previous studies reported rates of HF in HCM between 5.3 and 14/1000 patient years [5, 22, 23, 32]. The incidence in our cohort was slightly higher (20/1000 patient years), which is likely to reflect our older patient population. Use of medications to reduce development and progression of HF in HCM has been proposed [33] but trial data are lacking.

For trial design, use of surrogate endpoints with probable mechanistic link to HF may improve trial feasibility and cost, since the annual incidence of HF events in HCM is low. We suggest that predictors of HF that remain significant on multivariable analysis, and have a plausible mechanistic relationship with outcome, such as ventricular replacement fibrosis, may be suitable surrogate outcome measures.

In keeping with previous work, we found that patients who subsequently developed HF had a larger LV cavity and a larger LA volume at baseline [8, 22]. Interestingly, we did not find presence of LVOT obstruction to be predictive of HF, in contrast to a large prior cohort study [34]. Our findings were in keeping with Harris et al., where patients with progression to end-stage HF were more likely to have non-obstructive disease at baseline.

#### Clinical implications

Myocardial replacement fibrosis is a likely mechanism of progression to HF and was strongly predictive of future HF events. Visual myocardial perfusion score using CMR did not predict future HF, however quantitative perfusion using PET has previously been predictive. Development and testing of novel therapies to reduce progression of HF is an important unmet need in HCM. Trials are hampered by a low event rate. Use of surrogate endpoints with probable mechanistic link to HF, such as %LGE may improve trial feasibility and cost.

#### Study limitations

Although consecutive patients were enrolled, the study design has potential for referral bias. Patients referred for CMR may have been more symptomatic or of clinical concern. Patients with an ICD or pacemaker were excluded as these were relative contraindications to CMR, which may have excluded high risk patients.

Our HF endpoint was largely driven by progression to NYHA class III/IV rather than HF death or transplantation. As event rates of HF were low, statistical power for multivariable analyses was limited.

We used visual assessment of myocardial perfusion rather than absolute myocardial perfusion assessment using CMR. This is because we did not acquire an arterial input function for all patients [20]. Quantitative myocardial perfusion correlated moderately with the visual perfusion score and results may have been different had perfusion been fully quantitated. In addition, visual perfusion analysis did not exclude regions of replacement fibrosis, which may have altered the findings.

T1 mapping was not available at the outset of the study and therefore not performed, however would have given a measure of global interstitial fibrosis. LVOT obstruction was assessed at rest but provocation for latent obstruction was not performed.

The mean age of our patients was 60 years and there was a higher prevalence of the apical variant compared to other studies. Our population had higher rates of hypertension and diabetes than previous studies which are known to cause microvascular disease; however, this is reflective of real-life practice. There may have been unrecognized underlying coronary artery disease. We did not include pediatric patients. We did not systematically measure brain natriuretic peptide which may have given additional prognostic information.

## Conclusions

We prospectively assessed clinical and CMR predictors of HF in the HCM population. LVESVI and %LGE were the strongest predictors of HF. CMR visual assessment of myocardial perfusion did not predict HF in our cohort.

## Data Availability

The datasets generated and or analysed during the current study are not publicly available due to ongoing research but are available from the corresponding author on reasonable request.

## Abbreviations

AHA: American Heart Association
BSA: Body surface area
CMR: Cardiovascular magnetic resonance
FWHM: Full width half maximum
HCM: Hypertrophic cardiomyopathy
HF: Heart failure
ICD: Implanted cardiodefibrillator
LA: Left atrium/left atrial
LAVI: Left atrial volume indexed to body surface area
LGE: Late gadolinium enhancement
LV: Left ventricle/left ventricular
LVEDVI: Left ventricular end-diastolic index
LVEF: Left ventricular ejection fraction
LVESVI: Left ventricular end systolic volume indexed to body surface area
LVOT: Left ventricular outflow tract
MBF: Myocardial blood flow
MPRI: Myocardial perfusion reserve index
NYHA: New York Heart Association
PET: Positron emission tomography
SCD: Sudden cardiac death
SDS: Sum difference score
SPECT: Single photon emission computed tomography
